# Supplementary material for: Evaluating the effectiveness of care coordination interventions designed and implemented through a participatory action research process: Lessons learned from a quasi-experimental study in public healthcare networks in Latin America
Source: PLoS One. 2022 Jan 12;17(1):e0261604. doi: 10.1371/journal.pone.0261604 (PMC8754346; doi:10.1371/journal.pone.0261604)
Supplement: S1 Table — (DOCX) [file pone.0261604.s001.docx]

**S1 Table.** Demographic, employment and organizational characteristics of the baseline survey sample (2015), stratified by country and network

|  | **Brazil** | | **Chile** | | **Colombia** | | **Mexico** | | **Uruguay** | |
| --- | --- | --- | --- | --- | --- | --- | --- | --- | --- | --- |
|  | **Intervention network** | **Control network** | **Intervention network** | **Control network** | **Intervention network** | **Intervention network** | **Control network** | **Intervention network** | **Control network** | **Intervention network** |
|  | **180** | **201** | **173** | **175** | **181** | **182** | **184** | **181** | **178** | **175** |
|  | **n(%)** | **n(%)** | **n(%)** | **n(%)** | **n(%)** | **n(%)** | **n(%)** | **n(%)** | **n(%)** | **n(%)** |
| **Sex** |  |  |  |  |  |  |  |  |  |  |
| Male | 96 (53.33) | 65 (32.34) | 93 (53.76) | 89 (50.86) | 108 (59.67) | 129 (54.14) | 104 (56.52) | 98 (54.14) | 75 (42.13) | 86 (49.14) |
| Female | 84 (46.67) | 136 (67.66) | 80 (46.24) | 86 (49.14) | 73 (40.33) | 53 (29.12) | 80 (43.48) | 83 (45.86) | 103 (57.87) | 88 (50.29) |
| **Age** |  |  |  |  |  |  |  |  |  |  |
| 23 to 35 years | 57 (31.67) | 47 (23.38) | 66 (38.15) | 67 (38.29) | 102 (56.35) | 87 (47.80) | 15 (8.15) | 23 (12.71) | 36 (20.22) | 24 (13.71) |
| 36 to 50 years | 85 (47.22) | 70 (34.83) | 65 (37.57) | 61 (34.86) | 41 (22.65) | 53 (29.12) | 69 (37.50) | 70 (38.67) | 86 (48.31) | 80 (45.71) |
| 51 to 77 years | 38 (21.11) | 80 (39.80) | 42 (24.28) | 47 (26.86) | 38 (20.99) | 40 (21.98) | 100 (54.35) | 88 (48.62) | 54 (30.34) | 67 (38.29) |
| **Healthcare level** |  |  |  |  |  |  |  |  |  |  |
| Primary care | 58 (32.22) | 51 (25.37) | 71 (41.04) | 70 (40.00) | 66 (36.46) | 52 (28.57) | 86 (46.74) | 70 (38.67) | 49 (27.53) | 60 (34.29) |
| Secondary care | 122 (67.78) | 150 (74.63) | 102 (58.96) | 105 (60.00) | 115 (63.54) | 130 (71.43) | 98 (53.26) | 111 (61.33) | 129 (72.47) | 115 (65.71) |
| **Time working at the center** |  |  |  |  |  |  |  |  |  |  |
| ≤ 1 year | 39 (21.67) | 34 (16.92) | 68 (39.31) | 49 (28.00) | 65 (35.91) | 55 (30.22) | 11 (5.98) | 16 (8.84) | 16 (8.99) | 19 (10.86) |
| > 1 year to 3 years | 67 (37.22) | 45 (22.39) | 32 (18.50) | 29 (16.57) | 57 (31.49) | 41 (22.53) | 29 (10.87) | 20 (11.05) | 32 (17.98) | 22 (12.57) |
| > 3 years | 74 (41.11) | 122 (60.70) | 73 (42.20) | 97 (55.43) | 59 (32.60) | 86 (47.25) | 153 (83.15) | 145 (80.11) | 130 (73.03) | 134 (76.57) |
| **Type of contract** |  |  |  |  |  |  |  |  |  |  |
| Permanent | 123 (68.33) | 169 (84.08) | 48 (27.75) | 81 (46.29) | 40 (22.10) | 33 (18.13) | 147 (79.89) | 148 (81.77) | 136 (76.40) | 122 (69.71) |
| Temporary | 57 (31.67) | 32 (15.92) | 121 (69.94) | 92 (52.57) | 134 (74.03) | 149 (81.87) | 36 (19.57) | 33 (18.23) | 37 (20.79) | 46 (26.29) |
| **Contracted hours per week** |  |  |  |  |  |  |  |  |  |  |
| ≤ 20 hours | 65 (36.11) | 100 (49.75) | 20 (11.56) | 14 (8.00) | 23 (12.71) | 18 (9.89) | 1 (0.54) | 1 (0.55) | 104 (58.43) | 99 (56.57) |
| 20 to 40 hours | 98 (54.44) | 89 (44.28) | 76 (43.93) | 97 (55.43) | 48 (26.52) | 71 (39.01) | 174 (94.57) | 177 (97.79) | 58 (32.58) | 60 (34.29) |
| > 40 hours | 17 (9.44) | 12 (5.97) | 77 (44.51) | 64 (36.57) | 110 (60.77) | 93 (51.10) | 9 (4.89) | 3 (1.66) | 16 (8.99) | 16 (9.14) |
| **Working in private sector^a^** | 101 (56.11) | 110 (54.73) | 96 (55.49) | 104 (59.43) | 68 (37.78) | 63 (34.81) | 87 (47.28) | 91 (50.56) | 160 (90.91) | 146 (84.88) |
| **Time per patient *** |  |  |  |  |  |  |  |  |  |  |
| *Primary care* | 28 (48.28) | 26 (50.98) | 61 (85.92) | 42 (60.0) | 4 (6.06) | 2 (3.85) | 3 (3.49) | 6 (8.57) | 38 (77.55) | 50 (83.33) |
| 15 minutes or less | 30 (51.72) | 25 (49.02) | 10 (14.08) | 28 (40.0) | 62 (93.94) | 50 (96.15) | 83 (96.51) | 62 (88.57) | 9 (18.37) | 10 (16.67) |
| More than 15 minutes |  |  |  |  |  |  |  |  |  |  |
| *Secondary care* | 104 (85.25) | 111 (74.0) | 56 (54.90) | 67 (63.81) | 42 (36.52) | 33 (25.38) | 15 (15.31) | 22 (19.82) | 106 (82.17) | 92 (80.0) |
| 15 minutes or less | 17 (13.93) | 39 (26.0) | 44 (43.14) | 38 (36.19) | 72 (62.61) | 94 (72.31) | 78 (79.59) | 78 (70.27) | 23 (17.83) | 23 (20.0) |
| More than 15 minutes | 101 (56.11) | 110 (54.73) | 96 (55.49) | 104 (59.43) | 68 (37.78) | 63 (34.81) | 87 (47.28) | 91 (50.56) | 160 (90.91) | 146 (84.88) |
| **Enough time during consultation for clinical coordination ^b^** | 64 (36.57) | 75 (37.50) | 26 (15.03) | 23 (13.22) | 23 (12.71) | 38 (10.88) | 36 (19.89) | 59 (34.10) | 70 (42.42) | 67 (41.10) |
| ***** Prevalence calculated using the total for the corresponding healthcare level as denominator. | | | | | | | | | | |
| **^a^** Affirmative answers are shown. | | | | | | | | | | |
| **^b^** Always + often categories. | | | | | | | | | | |
|  | | | | | | | | | | |
