# Supplementary material for: Evaluating the effectiveness of care coordination interventions designed and implemented through a participatory action research process: Lessons learned from a quasi-experimental study in public healthcare networks in Latin America
Source: PLoS One. 2022 Jan 12;17(1):e0261604. doi: 10.1371/journal.pone.0261604 (PMC8754346; doi:10.1371/journal.pone.0261604)
Supplement: S2 Table — (DOCX) [file pone.0261604.s002.docx]

**S2 Table.** Changes in the influencing factors of cross-level clinical coordination (intermediate outcomes) between 2015 and 2017 in the control networks, by country

|  | **Brazil** | **Chile** | **Colombia** | **Mexico** | **Uruguay** |
| --- | --- | --- | --- | --- | --- |
|  | **CN 2015/2017** | **CN 2015/2017** | **CN 2015/2017** | **CN 2015/2017** | **CN 2015/2017** |
|  | **PR (IC 95%)*** | **PR (IC 95%)*** | **PR (IC 95%)*** | **PR (IC 95%)*** | **PR (IC 95%)*** |
| ***Interactional factors between professionals*** |  |  |  |  |  |
| Knowing the doctors of the other care level personally | 1.42 (0.82-2.47) | 1.11 (0.49-2.48) | 1.43 (0.86-2.40) | 1.43 (0.88-2.31) | 0.99 (0.85-1.15) |
| Trusting in clinical skills of doctors of the other care level | 1.13 (0.94-1.35) | **1.20 (1.02-1.41)** | **1.20 (1.02-1.41)** | 1.11 (0.94-1.32) | 0.98 (0.89-1.08) |
| Identification of PC doctors as coordinators of patient care across care levels | 1.11 (0.93-1.33) | 1.03 (0.87-1.21) | 1.05 (0.85-1.28) | 1.16 (0.99-1.35) | 0.96 (0.81-1.14) |
| ***Organizational factors*** |  |  |  |  |  |
| PC centre managers facilitate clinical coordination between care levels | 1.10 (0.72-1.69) | 1.23 (0.87-1.74) | **2.09 (1.36-3.23)** | 1.06 (0.77-1.46) | 1.19 (0.90-1.58) |
| SC centre managers facilitate clinical coordination between care levels | 0.99 (0.65-1.50) | 1.18 (0.76-1.82) | **1.64 (1.12-2.41)** | 1.35 (0.95-1.91) | 1.18 (0.88-1.58) |

* Adjusted for: sex, age, healthcare level. CN: control network. PR: prevalence ratio. PC: primary care. SC: secondary care.
