# Supplementary material for: Evaluating the effectiveness of care coordination interventions designed and implemented through a participatory action research process: Lessons learned from a quasi-experimental study in public healthcare networks in Latin America
Source: PLoS One. 2022 Jan 12;17(1):e0261604. doi: 10.1371/journal.pone.0261604 (PMC8754346; doi:10.1371/journal.pone.0261604)
Supplement: S3 Table — (DOCX) [file pone.0261604.s003.docx]

**S3 Table.** Differences in influencing factors of cross-level clinical coordination (intermediate outcomes) in 2015 in the intervention and control networks, by country

|  | **Brazil** | **Chile** | **Colombia** | **Mexico** | **Uruguay** |
| --- | --- | --- | --- | --- | --- |
|  | **IN vs. CN 2015** | **IN vs. CN 2015** | **IN vs. CN 2015** | **IN vs. CN 2015** | **IN vs. CN 2015** |
|  | **PR (IC 95%)** | **PR (IC 95%)** | **PR (IC 95%)** | **PR (IC 95%)** | **PR (IC 95%)** |
| ***Interactional factors between professionals*** |  |  |  |  |  |
| Knowing the doctors of the other care level personally | 1.45 (0.80-2.62) | **2.33 (1.14-4.76)** | **0.30 (0.12-0.72)** | 0.92 (0.53-1.59) | 1.11 (0.97-1.27) |
| Trusting in clinical skills of doctors of the other care level | 1.10 (0.91-1.34) | 1.19 (1.00-1.42) | 1.04 (0.88-1.23) | 1.05 (0.87-1.24) | 1.05 (0.95-1.16) |
| Identification of PC doctors as coordinators of patient care across care levels | 1.10 (0.92-1.33) | 1.09 (0.91-1.30) | 1.07 (0.88-1.29) | 0.97 (0.84-1.13) | 0.86 (0.71-1.05) |
| ***Organizational factors*** |  |  |  |  |  |
| PC centre managers facilitate the clinical coordination between care levels | 1.18 (0.76-1.85) | 0.92 (0.62-1.37) | 1.20 (0.74-1.97) | **0.58 (0.39-0.85)** | 0.88 (0.63-1.22) |
| SC centre managers facilitate the clinical coordination between care levels | 1.37 (0.91-2.06) | 1.02 (0.65-1.62) | 0.95 (0.61-1.50) | 0.91 (0.61-1.35) | 1.01 (0.74-1.38) |

* Adjusted for: sex, age, healthcare level. IN: intervention network. CN: control network. PR: prevalence ratio. PC: primary care. SC: secondary care
