# Supplementary material for: Evaluating the effectiveness of care coordination interventions designed and implemented through a participatory action research process: Lessons learned from a quasi-experimental study in public healthcare networks in Latin America
Source: PLoS One. 2022 Jan 12;17(1):e0261604. doi: 10.1371/journal.pone.0261604 (PMC8754346; doi:10.1371/journal.pone.0261604)
Supplement: S4 Table — (DOCX) [file pone.0261604.s004.docx]

**S4 Table.** Changes in the experience of cross-level coordination of information and clinical management of care (distal outcomes) between 2015 and 2017 in the control networks, by country

|  | **Brazil** | **Chile** | **Colombia** | **Mexico** | **Uruguay** |
| --- | --- | --- | --- | --- | --- |
|  | **CN 2015/2017** | **CN 2015/2017** | **CN 2015/2017** | **CN 2015/2017** | **CN 2015/2017** |
|  | **PR (IC 95%)** | **PR (IC 95%)** | **PR (IC 95%)** | **PR (IC 95%)** | **PR (IC 95%)** |
| ***Coordination of information*** |  |  |  |  |  |
| Exchange of information between care levels | 0.92 (0.63-1.35) | 0.99 (0.54-1.81) | 1.18 (0.99-1.41) | 1.38 (0.87-2.18) | 0.87 (0.67-1.13) |
| ***Consistency of care across care levels*** |  |  |  |  |  |
| Agreement over the treatments prescribed by the other care level | 0.97 (0.78-1.20) | 1.06 (0.91-1.23) | 1.20 (0.99-1.47) | **1.28 (1.04-1.57)** | 1.21 (0.97-1.50) |
| Contradictions and/or duplications in the treatments prescribed by different care levels | 1.01 (0.72-1.66) | 1.10 (0.72-1.69) | 0.80 (0.54-1.20) | 1.26 (0.79-2.01) | 0.65 (0.39-1.09) |
| Repetition of tests that were already performed at the other care level | 1.24 (0.80-1.90) | 1.22 (0.93-1.60) | 0.72 (0.51-1.00) | 1.23 (0.88-1.72) | 1.09 (0.61-1.93) |
| PC refers the patient to SC when necessary | 1.08 (0.97-1.19) | 1.12 (0.99-1.27) | 0.99 (0.89-1.09) | 0.99 (0.88-1.12) | 0.99 (0.90-1.11) |
| ***Patient follow-up between care levels*** |  |  |  |  |  |
| SC doctors make recommendations to PC doctors for patient follow-up | 1.19 (0.89-1.58) | 0.97 (0.77-1.21) | 1.14 (0.93-1.39) | 1.01 (0.83-1.23) | 0.94 (0.75-1.16) |
| PC doctors consult SC doctors with any queries about patient follow-up | 1.40 (0.95-2.07) | 0.96 (0.53-1.71) | 1.07 (0.91-1.27) | 1.22 (0.70-2.10) | 1.07 (0.90-1.27) |
| SC refers patients to PC for follow-up | 0.91 (0.78-1.07) | 1.04 (0.88-1.23) | 0.99 (0.75-1.33) | 1.06 (0.90-1.24) | 0.93 (0.75-1.16) |

* Adjusted for: sex, age, healthcare level. CN: control network. PR: prevalence ratio. PC: primary care. SC: secondary care.
