# Supplementary material for: Evaluating the effectiveness of care coordination interventions designed and implemented through a participatory action research process: Lessons learned from a quasi-experimental study in public healthcare networks in Latin America
Source: PLoS One. 2022 Jan 12;17(1):e0261604. doi: 10.1371/journal.pone.0261604 (PMC8754346; doi:10.1371/journal.pone.0261604)
Supplement: S5 Table — (DOCX) [file pone.0261604.s005.docx]

**S5 Table.** Differences in experience of cross-level coordination of information and clinical management of care (distal outcomes) in 2015 in the intervention and control networks, by country

|  | **Brazil** | **Chile** | **Colombia** | **Mexico** | | **Uruguay** |
| --- | --- | --- | --- | --- | --- | --- |
|  | **IN vs. CN 2015** | **IN vs. CN 2015** | **IN vs. CN 2015** | | **IN vs. CN 2015** | **IN vs. CN 2015** |
|  | **PR (IC 95%)** | **PR (IC 95%)** | **PR (IC 95%)** | | **PR (IC 95%)** | **PR (IC 95%)** |
| ***Coordination of information*** |  |  |  | |  |  |
| Exchange of information between care levels | 1.15 (0.75-1.77) | 0.97 (0.53-1.78) | 1.18 (0.95-1.48) | | 0.96 (0.59-1.57) | 0.98 (0.78-1.25) |
| ***Consistency of care across care levels*** |  |  |  | |  |  |
| Agreement over the treatments prescribed by the other care level | 0.99 (0.79-1.23) | 0.90 (0.77-1.06) | 0.89 (0.71-1.12) | | 0.94 (0.75-1.17) | 1.02 (0.80-1.30) |
| Contradictions and/or duplications in the treatments prescribed by different care levels | **1.53 (1.02-2.30)** | 0.99 (0.63-1.56) | 1.08 (0.76-1.53) | | 1.23 (0.77-1.96) | 0.80 (0.50-1.30) |
| Repetition of tests that were already performed at the other care level | 1.26 (0.82-1.94) | 0.81 (0.59-1.12) | 1.19 (0.90-1.58) | | 0.94 (0.66-1.34) | 1.07 (0.60-1.91) |
| PC refers the patient to SC when necessary | 0.99 (0.88-1.11) | 1.08 (0.94-1.24) | 1.00 (0.91-1.10) | | 0.95 (0.85-1.08) | 1.01 (0.91-1.13) |
| ***Patient follow-up between care levels*** |  |  |  | |  |  |
| SC doctors make recommendations to PC doctors for patient follow-up | 0.78 (0.55-1.11) | 0.88 (0.70-1.12) | **0.77 (0.60-0.99)** | | 0.84 (0.68-1.04) | 0.99 (0.80-1.22) |
| PC doctors consult SC doctors with any queries about patient follow-up | 0.68 (0.41-1.12) | 1.11 (0.62-1.95) | **0.77 (0.63-0.95)** | | 0.57 (0.30-1.10) | 0.99 (0.83-1.19) |
| SC refers patients to PC for follow-up | 1.04 (0.89-1.22) | 1.00 (0.83-1.19) | 1.03 (0.78-1.35) | | 0.85 (0.71-1.03) | 0.98 (0.78-1.23) |

* Adjusted for: sex, age, healthcare level. IN: intervention network. CN: control network. PR: prevalence ratio. PC: primary care. SC: secondary care.
