# Supplementary material for: Evaluating the effectiveness of care coordination interventions designed and implemented through a participatory action research process: Lessons learned from a quasi-experimental study in public healthcare networks in Latin America
Source: PLoS One. 2022 Jan 12;17(1):e0261604. doi: 10.1371/journal.pone.0261604 (PMC8754346; doi:10.1371/journal.pone.0261604)
Supplement: S6 Table — (DOCX) [file pone.0261604.s006.docx]

|  | **Brazil** | | | | **Chile** | | | | **Colombia** | | | | **Mexico** | | | | **Uruguay** | | | | |
| --- | --- | --- | --- | --- | --- | --- | --- | --- | --- | --- | --- | --- | --- | --- | --- | --- | --- | --- | --- | --- | --- |
|  | **Intervention network** | | **Control network** | | **Intervention network** | | **Control network** | | **Intervention network** | | **Control network** | | **Intervention network** | | **Control network** | | **Intervention network** | | **Controlnetwork** | | |
|  | **2015** | **2017** | **2015** | **2017** | **2015** | **2017** | **2015** | **2017** | **2015** | **2017** | **2015** | **2017** | **2015** | **2017** | **2015** | **2017** | **2015** | **2017** | **2015** | **2017** |  |
|  | **n %** | **n %** | **n %** | **n %** | **n %** | **n %** | **n %** | **n %** | **n %** | **n %** | **n %** | **n %** | **n %** | **n %** | **n %** | **n %** | **n %** | **n %** | **n %** | **n %** |  |
| ***Interactional factors between professionals*** |  |  |  |  |  |  |  |  |  |  |  |  |  |  |  |  |  |  |  |  |  |
| Knowing the doctors of the other care level personally | 23 (12.78) | 39 (21.67) | 19 (9.45) | 26 (14.29) | 23 (13.29) | 18 (10.84) | 10 (5.71) | 12 (6.15) | 6 (3.31) | 18 (9.89) | 20 (10.99) | 33 (18.33) | 20 (10.87) | 26 (14.36) | 22 (12.15) | 37 (20.44) | 130 (73.03) | 121 (68.75) | 119 (68.0) | 115 (65.71) |  |
| Trusting in clinical skills of doctors of the other care level | 98 (54.44) | 118 (65.56) | 99 (49.25) | 105 (57.69) | 98 (56.65) | 98 (59.04) | 84 (48.00) | 120 (61.54) | 106 (58.56) | 110 (60.44) | 98 (53.85) | 118 (65.56) | 98 (53.26) | 89 (49.17) | 84 (46.41) | 107 (59.12) | 146 (82.02) | 143 (81.25) | 143 (81.71) | 141 (80.57) |  |
| Identification of PC doctors as coordinators of patient care across care levels | 105 (58.33) | 131 (72.78) | 97 (48.26) | 104 (57.14) | 103 (59.54) | 110 (66.27) | 93 (53.14) | 115 (58.97) | 101 (55.80) | 92 (50.55) | 88 (48.35) | 92 (51.11) | 103 (55.98) | 92 (50.83) | 92 (50.83) | 118 (65.19) | 86 (48.31) | 104 (59.09) | 100 (57.14) | 102 (58.29) |  |
| ***Organizational factors*** |  |  |  |  |  |  |  |  |  |  |  |  |  |  |  |  |  |  |  |  |  |
| PC centre managers facilitate clinical coordination between care levels | 36 (20.00) | 57 (31.67) | 31 (15.42) | 34 (18.68) | 33 (19.08) | 32 (19.28) | 37 (21.14) | 52 (26.67) | 30 (16.57) | 40 (21.98) | 23 (12.64) | 55 (30.56) | 31 (16.85) | 51 (28.18) | 48 (26.52) | 56 (30.94) | 43 (24.16) | 48 (27.27) | 50 (28.57) | 67 (38.29) |  |
| SC centre managers facilitate clinical coordination between care levels | 43 (23.89) | 48 (26.67) | 36 (17.91) | 33 (18.13) | 28 (16.18) | 22 (13.25) | 28 (16.0) | 34 (17.44) | 28 (15.47) | 39 (21.43) | 30 (16.48) | 55 (30.56) | 36 (19.57) | 66 (36.46) | 40 (22.10) | 56 (30.94) | 54 (30.34) | 62 (35.23) | 49 (28.00) | 62 (35.43) |  |

**S6 Table**. Distribution of influencing factors (intermediate outcomes) of clinical coordination between care levels, intervention and control networks in 2015 and 2017, by country

Categories were grouped into; yes = always and often; No = sometimes, rarely, never. Here the results for the first category (yes) are shown
