# Supplementary material for: Evaluating the effectiveness of care coordination interventions designed and implemented through a participatory action research process: Lessons learned from a quasi-experimental study in public healthcare networks in Latin America
Source: PLoS One. 2022 Jan 12;17(1):e0261604. doi: 10.1371/journal.pone.0261604 (PMC8754346; doi:10.1371/journal.pone.0261604)
Supplement: S7 Table — (DOCX) [file pone.0261604.s007.docx]

**S7 Table**. Distribution of experience of cross-level coordination of information and clinical management of care (distal outcomes), intervention and control networks in 2015 and 2017, by country

|  | **Brazil** | | | | **Chile** | | | | **Colombia** | | | | **Mexico** | | | | **Uruguay** | | | |
| --- | --- | --- | --- | --- | --- | --- | --- | --- | --- | --- | --- | --- | --- | --- | --- | --- | --- | --- | --- | --- |
|  | **Intervention network** | | **Control network** | | **Intervention network** | | **Control network** | | **Intervention network** | | **Control network** | | **Intervention network** | | **Control network** | | **Intervention network** | | **Control network** | |
|  | **2015** | **2017** | **2015** | **2017** | **2015** | **2017** | **2015** | **2017** | **2015** | **2017** | **2015** | **2017** | **2015** | **2017** | **2015** | **2017** | **2015** | **2017** | **2015** | **2017** |
|  | **n %** | **n %** | **n %** | **n %** | **n %** | **n %** | **n %** | **n %** | **n %** | **n %** | **n %** | **n %** | **n %** | **n %** | **n %** | **n %** | **n %** | **n %** | **n %** | **n %** |
| ***Consistency of care across care levels*** |  |  |  |  |  |  |  |  |  |  |  |  |  |  |  |  |  |  |  |  |
| Agreement over the treatments prescribed by the other care level | 135 (75.0) | 139 (77.22) | 148 (73.63) | 148 (81.32) | 119 (68.79) | 100 (60.24) | 110 (62.86) | 145 (74.36) | 149 (82.32) | 146 (80.22) | 145 (79.67) | 143 (79.44) | 120 (65.22) | 127 (70.17) | 113 (62.43) | 123 (67.96) | 136 (76.40) | 142 (80.68) | 140 (80.0) | 138 (78.86) |
| Contradictions and/or duplications in the treatments prescribed by different care levels | 35 (19.44) | 23 (12.78) | 32 (15.92) | 36 (19.78) | 45 (26.01) | 47 (28.31) | 56 (32.0) | 73 (37.44) | 63 (34.81) | 49 (26.92) | 57 (31.32) | 41 (22.78) | 40 (21.74) | 59 (32.60) | 47 (25.97) | 49 (27.07) | 22 (12.36) | 16 (9.09) | 23 (13.14) | 22 (12.57) |
| Repetition of tests that were already performed at the other care level | 83 (46.11) | 102 (56.67) | 87 (43.28) | 80 (43.96) | 81 (46.82) | 88 (53.01) | 88 (50.29) | 114 (58.46) | 74 (40.88) | 97 (53.30) | 81 (44.51) | 96 (53.33) | 68 (36.96) | 60 (33.15) | 61 (33.70) | 92 (50.83) | 75 (42.13) | 101 (57.39) | 76 (43.43) | 92 (52.57) |
| PC refers the patient to SC when necessary | 47 (26.11) | 36 (20.0) | 36 (17.91) | 36 (19.87) | 30 (17.34) | 35 (21.08) | 31 (17.71) | 39 (20.0) | 48 (26.52) | 35 (19.23) | 44 (24.18) | 34 (18.89) | 33 (17.93) | 35 (19.34) | 26 (14.36) | 33 (18.23) | 24 (13.48) | 19 (10.80) | 34 (19.43) | 20 (11.43) |
| ***Patient follow-up between care levels*** |  |  |  |  |  |  |  |  |  |  |  |  |  |  |  |  |  |  |  |  |
| SC doctors make recommendations to PC doctors for patient follow-up | 109 (60.56) | 110 (61.11) | 128 (63.68) | 108 (59.34) | 98 (56.65) | 105 (63.25) | 99 (56.57) | 118 (60.51) | 66 (36.46) | 51 (28.02) | 58 (31.87) | 58 (32.22) | 94 (51.09) | 103 (56.91) | 109 (60.22) | 114 (62.98) | 79 (44.38) | 84 (47.73) | 81 (46.29) | 79 (45.14) |
| PC doctors consult SC doctors with any queries about patient follow-up | 41 (22.78) | 52 (28.89) | 60 (29.85) | 63 (34.62) | 69 (39.88) | 88 (53.01) | 80 (45.71) | 85 (43.59) | 59 (32.60) | 62 (34.07) | 81 (44.51) | 95 (52.78) | 74 (40.22) | 97 (53.59) | 94 (51.93) | 91 (50.28) | 87 (48.88) | 81 (46.02) | 83 (47.43) | 81 (46.29) |
| SC refers patients to PC for follow-up | 22 (12.22) | 32 (17.78) | 35 (17.41) | 45 (24.73) | 22 (12.72) | 26 (15.66) | 20 (11.43) | 23 (11.79) | 71 (39.23) | 71 (39.01) | 98 (53.85) | 106 (58.89) | 12 (6.52) | 25 (13.81) | 21 (11.60) | 24 (13.26) | 100 (56.18) | 101 (57.39) | 100 (57.14) | 111 (63.43) |

Categories were grouped into; yes = always and often; No = sometimes, rarely, never. Here the results for the first category (yes) are shown
