# Supplementary material for: Evaluating the effectiveness of care coordination interventions designed and implemented through a participatory action research process: Lessons learned from a quasi-experimental study in public healthcare networks in Latin America
Source: PLoS One. 2022 Jan 12;17(1):e0261604. doi: 10.1371/journal.pone.0261604 (PMC8754346; doi:10.1371/journal.pone.0261604)
Supplement: S8 Table — (DOCX) [file pone.0261604.s008.docx]

**S8 Table**. Perception of coordination between care levels (distal outcomes), in the intervention and control networks in 2015 and 2017, by country

|  | **Brazil** | | | | **Chile** | | | | **Colombia** | | | | **Mexico** | | | | **Uruguay** | | | | |
| --- | --- | --- | --- | --- | --- | --- | --- | --- | --- | --- | --- | --- | --- | --- | --- | --- | --- | --- | --- | --- | --- |
|  | **Intervention network** | | **Control network** | | **Intervention network** | | **Control network** | | **Intervention network** | | **Control network** | | **Intervention network** | | **Control network** | | **Intervention network** | | | **Control network** | |
|  | **2015** | **2017** | **2015** | **2017** | **2015** | **2017** | **2015** | **2017** | **2015** | **2017** | **2015** | **2017** | **2015** | **2017** | **2015** | **2017** | | **2015** | **2017** | **2015** | **2017** |
|  | **n %** | **n %** | **n %** | **n %** | **n %** | **n %** | **n %** | **n %** | **n %** | **n %** | **n %** | **n %** | **n %** | **n %** | **n %** | **n %** | | **n %** | **n %** | **n %** | **n %** |
| General perception of clinical coordination between care levels | 16 (8.89) | 32 (17.78) | 7 (3.48) | 15 (8.24) | 23 (13.29) | 19 (11.45) | 19 (10.86) | 28 (14.36) | 46 (25.41) | 64 (35.16) | 42 (23.08) | 103 (57.22) | 16 (8.70) | 39 (21.55) | 32 (17.68) | 40 (22.10) | | 56 (31.46) | 59 (33.52) | 58 (33.14) | 51 (29.14) |

Categories were grouped into; yes = always and often; No = sometimes, rarely, never. Here the results for the first category (yes) are shown.
