# Supplementary material for: Evaluating the effectiveness of care coordination interventions designed and implemented through a participatory action research process: Lessons learned from a quasi-experimental study in public healthcare networks in Latin America
Source: PLoS One. 2022 Jan 12;17(1):e0261604. doi: 10.1371/journal.pone.0261604 (PMC8754346; doi:10.1371/journal.pone.0261604)
Supplement: S9 Table — (DOCX) [file pone.0261604.s009.docx]

**S9 Table. Summary of positive effects of PAR interventions on clinical coordination, by country: improvement in intermediate and distal outcomes of clinical coordination in IN between 2015 and 2017**

|  | **Brazil** | **Chile** | **Colombia** | **Mexico** | **Uruguay** |
| --- | --- | --- | --- | --- | --- |
| **Influencing factors of cross-level clinical coordination (intermediate outcomes)** | | | | | |
| ***Interactional factors between professionals*** | | | | | |
| Knowing the doctors of the other care level personally | ✓ |  |  |  |  |
| Trusting in clinical skills of doctors of the other care level | ✓ |  |  |  |  |
| Identification of PC doctors as coordinators of patient care across care levels | ✓ |  |  |  |  |
| ***Organizational factors*** | | | | | |
| PC centre managers facilitate clinical coordination between care levels |  |  |  | ✓ |  |
| SC centre managers facilitate clinical coordination between care levels |  |  | ✓ | ✓ |  |
| **Experience of cross-level coordination of information and clinical management of care (distal outcomes)** | | | | | |
| ***Coordination of information*** | | | | | |
| Exchange of information between care levels |  |  |  |  |  |
| ***Consistency of care across care levels*** | | | | | |
| Agreement over the treatments prescribed by the other care level | ✓ |  | ✓ |  | ✓ |
| Contradictions and/or duplications in the treatments prescribed by different care levels |  |  |  |  |  |
| Repetition of tests that were already performed at the other care level |  |  |  |  |  |
| PC refers the patient to SC when necessary |  |  |  | ✓ |  |
| ***Patient follow-up between care levels*** | | | | | |
| SC doctors make recommendations to PC doctors for patient follow-up |  | ✓ |  | ✓ |  |
| PC doctors consult SC doctors with any queries about patient follow-up |  |  |  | ✓ |  |
| **General perception of coordination between care levels (distal outcome)** | | | | | |
| Perception that care provided is coordinated between care levels | ✓ |  | ✓ | ✓ |  |

* Only items that improved with statistical significance are shown. IN: intervention network. PC: primary care. SC: secondary care
