# Supplementary material for: Evaluating the effectiveness of care coordination interventions designed and implemented through a participatory action research process: Lessons learned from a quasi-experimental study in public healthcare networks in Latin America
Source: PLoS One. 2022 Jan 12;17(1):e0261604. doi: 10.1371/journal.pone.0261604 (PMC8754346; doi:10.1371/journal.pone.0261604)
Supplement: Attachment — Submitted filename: RESPONSE TO REVIEWERS_Effectiveness PAR LA.doc [file pone.0261604.s010.doc]

**Editor’s comments**

*1. Please ensure that your manuscript meets PLOS ONE's style requirements, including those for file naming.*

We have revised the manuscript to ensure that it meets PLOS ONE’s style requirements.

*2. We note that you have indicated that data from this study are available upon request. PLOS only allows data to be available upon request if there are legal or ethical restrictions on sharing data publicly. In your revised cover letter, please address the following prompts:*

*a) If there are ethical or legal restrictions on sharing a de-identified data set, please explain them in detail (e.g., data contain potentially identifying or sensitive patient information) and who has imposed them (e.g., an ethics committee). Please also provide contact information for a data access committee, ethics committee, or other institutional body to which data requests may be sent.*

*b) If there are no restrictions, please upload the minimal anonymized data set necessary to replicate your study findings as either Supporting Information files or to a stable, public repository and provide us with the relevant URLs, DOIs, or accession numbers. Please see http://www.bmj.com/content/340/bmj.c181.long for guidelines on how to de-identify and prepare clinical data for publication. For a list of acceptable repositories, please see [http://journals.plos.org/plosone/s/data-availability#loc-recommended-repositories](http://journals.plos.org/plosone/s/data-availability#loc-recommended-repositories). We will update your Data Availability statement on your behalf to reflect the information you provide.*

We have held an in-depth discussion on the possibility of publishing the data on which this study is based with the Data Protection Officer of the project, and have concluded that there are no ethical or legal restrictions on sharing the data set publicly, since it is fully anonymous and presents minimal risk to the confidentiality of study participants.

We have updated our Data Availability statement to reflect this information and provided the URL of the public repository from which the data set can be downloaded.

*3. One of the noted authors is a group; Equity LA II. In addition to naming the author group, please list the individual authors and affiliations within this group in the acknowledgments section of your manuscript. Please also indicate clearly a lead author for this group along with a contact email address.*

We have listed the individual authors and affiliations within Equity-LA II, which contributed to the development of this study, as well as the lead author (María-Luisa Vázquez), in the acknowledgments section of the manuscript.

**Reviewer's Responses to Questions**

*1. Is the manuscript technically sound, and do the data support the conclusions?
The manuscript must describe a technically sound piece of scientific research with data that supports the conclusions. Experiments must have been conducted rigorously, with appropriate controls, replication, and sample sizes. The conclusions must be drawn appropriately based on the data presented.*

*Reviewer #1: Yes*

*Reviewer #2: Partly*

We have rewritten the conclusions in the abstract and discussion sections of the manuscript to emphasize the results more clearly, as indicated below.

*2. Have the authors made all data underlying the findings in their manuscript fully available?The [PLOS Data policy](http://www.plosone.org/static/policies.action#sharing) requires authors to make all data underlying the findings described in their manuscript fully available without restriction, with rare exception (please refer to the Data Availability Statement in the manuscript PDF file). The data should be provided as part of the manuscript or its supporting information, or deposited to a public repository.*

*Reviewer #1: Yes*

*Reviewer #2: No*

As stated under Editors’ comments 2, the data are now fully available from a public repository.

**Reviewer 1**

Firstly, we would like to thank the reviewer for their comments and the suggestions received, which we have incorporated into the revised manuscript.

1. *Please provide a definition or example of “levels of care” early in the paper. This would be helpful to the many PLOS One readers with limited healthcare experience. The terms “primary” and “secondary” care are not mentioned until page 6.*

“Levels of care” refers to primary care and secondary care, distinguished according to the level of complexity. We have now clarified the term “levels of care” earlier in the paper (line 91, page 4)

2. *Please also consider adding a very brief summary of the health systems/healthcare payment systems in the participating countries. Alternatively, this information could be added to Supplementary Table 1. Payment policy differences significantly influence coordination methods and time invested in coordination activities. These issues are mentioned briefly in the discussion section but should be addressed earlier.*

Following the reviewer’s recommendation, we have added a brief summary of the characteristics of the public healthcare sub-systems in the participating countries, including the health system funding and payment mechanisms). This will contribute to a better understanding of the context and the economic incentives to take part in the participatory process and the adoption of interventions (line 216, page 8).

3. *Please clarify how networks were identified as either an intervention or control network. I understand this was not random assignment, but did network leaders choose if they wanted to be in the intervention group and others did not? Was there some other way the “intervention” networks were selected or identified?*

The selection of networks in each country was not random, but was done by applying the following selection criteria: a) provision of a continuum of services to a defined population including at least primary and secondary care; b) mainly in urban areas of low or medium-low socioeconomic status; and c) willingness to participate and implement the designed interventions. Therefore, the selected intervention network had to be willing to implement the intervention, as stated in the last criterion. The participation of the networks was voluntary and there were no refusals to participate. The final decision was made by the researchers together with the managers of the networks.

We have added this information in the section on study design and study areas (line 248, page 9).

4. *Also, the networks differed in the design approaches used, implementation timelines, and types of interventions selected. It would seem that some subgroup analyses of the 3 networks with more similar design and implementation approaches could be useful (i.e.,Columbia, Brazil, and Mexico).*

As the reviewer points out, the participatory process, the type and characteristics of the interventions implemented, and the contextual factors that influenced the process differed across the five experiences. These aspects have been analyzed comparatively in two previous publications(1,2) and we are currently preparing a third article that focuses on a comparative analysis of the PAR processes for the design of the interventions in the study countries. Furthermore, several papers are underway that analyze in greater depth and detail the design and implementation process carried out in each country on an individual basis.

With regard to potential subgroups of analysis, although we acknowledge that there are some similarities, these are not enough to clearly establish groups of countries for comparative analysis: firstly, the experiences may have certain elements in common in terms of the participatory process, but they differ in several others, particularly in the type of interventions that were implemented and in the contextual factors that influenced them; and secondly, the number of study countries (five) is too small to be able to carry out a subgroup analysis.

5. *I commend the authors for the description of their conceptual model, providing definitions of key study terminology, and using an established survey instrument with prior psychometric evaluation data.*

We would like to thank the reviewer for the comments on the description of the conceptual model and survey instrument.

6. *Please clarify the sampling method (i.e., how were participating physicians identified and contacted to participate?) and provide response rates for the baseline and follow-up surveys. Also please clarify the proportion of respondents who completed both baseline and follow-up surveys. It should be clear if this is multiple time-point cross-sectional survey or a true pre-post survey of the same respondents.*

Following the reviewer's recommendation, we have added information about how the participants were contacted, and the response rate for both the baseline and the evaluation surveys in the data collection section. The selection of the participants for each survey was carried out based on the list of physicians working at the center at the time of survey who met the selection criteria. Therefore the participating physicians were not necessarily the same for both surveys (line 262, page 9)

A question was included in the evaluation survey (in some countries) asking about participation in the first survey (self-reported), since the survey was anonymous. We didn’t consider it appropriate to include this variable in the analysis, nor to report it in the article, because a recall bias was observed.

7. *Variables section, pg. 9: Perhaps consider using the terminology “intermediate outcomes” and “distal outcomes” rather than “intermediate results” and “final results” to describe these variables.*

Following the reviewer’s recommendation, we have replaced the term intermediate/final results with intermediate/distal outcomes consistently throughout the manuscript.

8. *Clarify why prevalence ratios were used. This suggests that survey item scores were dichotomized.*

The outcome variables were dichotomized by merging original answer options into: high (always/often=1) or low (sometimes/rarely/never=0). We have included this information in the methods section (line 331, page 10).

Thanks to the reviewer's comment, we realized that in the supplementary tables we did not include the corresponding table footer to indicate the grouping of the responses shown in the table. We have now added it.

9. *Sections of Supplementary Table 1 appear to be missing. If specific sections of the table are not applicable to particular countries it would be useful to note “not applicable” in the appropriate cells.*

Indeed, specific sections of the table are not applicable to particular countries. We have now added “NA = not applicable” in the corresponding cells (Table S1).

10. *Supplemental table 1 and Table note for Table 1 in the paper: Please clarify intervention 2 in Chile. It is currently described as “cross-level bidirectional visits,” but that description is not clear. Were these in-person multi-disciplinary visits where a patient would see both primary and secondary care providers in the same visit or on the same day? Or something else?*

The cross-level bidirectional visits were carried out by PC and SC teams. That is to say, both teams would visit the centres where the team of the other level worked. The objective was to promote “mutual knowledge” between professionals across care levels (i.e. knowledge of their work environment, getting to know each other in person, and establishing ties). Patients were not involved. The visits formed part of the network induction program for newly appointed personnel, alongside graphic and audiovisual material providing information on the network's characteristics (operational units, equipment, workers, population served, etc.) and activities. The goal of the induction program was to promote a common identity and shared vision in the healthcare network. The cross-level visits lasted 4 hours (half a work day), and a total of 4 visits were carried out during the project.

The details of the interventions are provided and analyzed in the article on the design of the interventions that we are currently preparing. However, we have added some more information in the Tables 1 and S1, and text (line 396, page 12) to clarify the contents of this intervention.

11. *The abstract and discussion section both emphasize the few statistically significant results from the large number of analyses conducted. Conclusions presented in the abstract in particular should be tempered with the limitations of the study and focus on the need for more robust evaluation approaches that are described in the discussion section.*

We have revised the conclusions in the abstract and the introduction to the discussion section to explain that: 1) the observed improvements refer to some items of clinical coordination; 2) there are differences between countries in the outcomes obtained; 3) a longer evaluation time frame is required to detect more quantifiable changes (line 65, page 3 and line 501-9, page 23). However, we would like to highlight, as indicated in the discussion, the importance of the achievements considering the limited time frame of this intervention evaluation and their consistency with the expected results of these interventions, which were selected by the health professionals themselves to improve their daily practice.

12. *Overall, sentence structure (particularly in the discussion/conclusions section) could be simplified and streamlined. Key points are hidden and hard to follow given the use of very long, complex sentences. This problem is easily correctable, but important if readers are to grasp key conclusions.*

*E.g., pg. 26, lines 502 to 508: The example below is a single sentence…though it reads like a paragraph…. “However, detecting changes at network/organization level poses challenges that have been extensively highlighted in fields such as community-based evaluation [51,52], such as low statistical power to detect differences because of the limited number of intervention and control organizations/networks that make up the sample, this being due to the financial and logistical difficulties/costs involved in increasing their number; or the need for a longer time frame for implementation, and thus more resources, to achieve greater penetration of the intervention and make an impact at organization/area level.”*

Following the reviewer's recommendations, we have revised the discussion and conclusions to simplify the sentence structures and clarify the main messages.

**Reviewer 2**

First of all, we would like to thank the reviewer for their comments and the suggestions received, which we have incorporated into the revised manuscript.

1. *Based on my reading of the manuscript, the authors appear to be evaluating the effectiveness of the PAR approach as an implementation intervention to improve coordination between primary and specialty care. However, the specific components of PAR are that were used in each country were not clearly reported, nor were the resulting changes taken by each country as a result of the PAR approach. In other words, PAR itself does not directly change coordination, it opens the door for the networks to identify the interventions (and implementation strategy) that they will need to change coordination. Those intermediate outcomes were not reported in the paper.*

Indeed, as stated by the reviewer, “*the PAR itself does not directly change coordination, but it opened the door for the networks to identify the interventions”* needed, and *“the intermediate results are not reported here”.*

In this regard, we would like to highlight that this study is only one part of a wider implementation research project (Equity-LA II) (3), which adopts a PAR approach at different levels and, as the reviewer highlights, particularly for the selection, design and implementation of specific interventions to improve cross-level clinical coordination. To evaluate the implemented interventions, this research combines qualitative data collection methods focusing on the design and implementation process of the interventions (5) with quantitative methods to analyze the effectiveness of the implemented intervention in terms of improvements in doctors’ care coordination experience at network level. The latter is the focus of this paper.

As the results of these exhaustive evaluations are so extensive, both for each country case and across countries, their analysis has been broken down into several different publications. The comparative analysis of the qualitative evaluation results focused on: 1) analysis of contextual factors and the PAR process that influenced the implementation of the interventions(1); 2) the results of the interventions in improving coordination between levels of care as perceived by the directly involved participants (2); and 3) analysis of the participatory process for the design of the interventions (in preparation). In other words, the intermediate outcomes of the PAR process indicated by the reviewer were evaluated with the participants of the interventions using qualitative research methods. The results have been published in two previous articles (2,5), and the third one is underway. These findings have been taken into account for the interpretation of the results concerning the effect of the intervention on cross-level care coordination in the discussion of the submitted manuscript.

With regard to the effect of the PAR process on care coordination, as mentioned above, the participatory process was aimed at designing and implementing interventions to improve coordination in the networks. In this process, the professionals who participated selected significant coordination problems in their daily practice, and subsequently selected and designed interventions to address these problems (6). Therefore, the objectives of the interventions were to improve care coordination between care levels. One of the results of the implementation process evaluation is that the participatory process, in some countries, not only contributed to a higher uptake of the interventions (by fostering professionals’ interest and motivation to participate), but also improved the interactional factors that influence coordination between levels of care (personal relationships, mutual trust and training of professionals) (2,5). In short, with differences between countries, the PAR process contributed directly to improving coordination between the participants of the interventions, as discussed in lines 570-7, page 25.

In order to clarify these points, we have provided further information about the qualitative evaluation and the references to related publications in the introduction of the article (lines 169- 182, page 6-7). We have also revised the discussion to better explain the contribution of the PAR process to improving coordination between levels (lines 570-7, page 25).

2. *In the discussion, the authors present an expansive section on the methodological challenges to participatory action research -- was this intended to serve as the limitations section of their study? If so, the section does not achieve its goal, as it does not report on the specific challenges of the study and how they were mitigated, but rather talks about general methodological challenges. If the intent of that section was not to serve as the limitations section, then the limitations section of the study is missing. Either way, specifics about the limitations of the study and attempt to mitigate these limitations is missing from the manuscript.*

This section details the methodological difficulties encountered over the course of this study and the strategies used to deal with them. Most of the difficulties are inherent to the nature of the PAR method, and have been reported by other community-based evaluations, but not in the field of health services evaluation. For this reason, it seemed important to us to include more generalized reflections and make recommendations for future evaluations of the effectiveness of PAR interventions in health services. We agree with the reviewer that some of these reflections are too general, and as a result, we have change the title of the section and re-rewritten the section to clarify the methodological difficulties found in this study, the strategies used to tackle them, and the recommendations for future evaluations (lines 639-826, page 27-28).

3. *There is a lot of confusion in the methods section in reporting of what was actually done. It took me to the end of the methods section to realize and understand that interactional factors, organizational factors, and the various clinical coordination factors were actually subscales in the COORDENA questionnaire. There's also talk throughout the paper about a qualitative study that is not reported in the manuscript. Either delete the language about the qualitative study, or re-ported in greater detail here.*

We have revised the methods section to make it clearer that the variables related to the final and intermediate outcomes of the interventions were collected using the COORDENA questionnaire. However, these are not subscales of the questionnaire, but items that are treated individually. Although detailed information on the design, structure, and validation of the questionnaire has already been published (4,7), we have also added some more information regarding the structure of the questionnaire (line 307, page 10).

With regard to the last point, as mentioned above, this study is part of a wider implementation research project (3) which combines qualitative research methods to evaluate the design and implementation process of the interventions (5) with quantitative methods to analyze their effectiveness in terms of care coordination. This comprehensive hybrid approach is increasingly adopted to simultaneously evaluate the clinical effectiveness of an intervention and the implementation process (8,9), to provide information on contextual effectiveness. In this paper, the qualitative evaluation of the implementation process, the factors that influenced it and the results perceived by participants, complements and facilitates interpretation of the findings of the effectiveness analysis. It helps to identify and understand the factors of success or failure for the intervention related to the context and the process, the mechanisms by which the intervention has contributed to the associated results, and other outcomes identified by the participants of the interventions but not detected in the evaluation in the whole network of health services. For this reason, they have been taken into account in the interpretation of the results in the discussion of the article.

In the introduction to the article we have now provided more information about the qualitative evaluation, references to previous publications, and clarified the results presented in this article (line 168, page 6).

4. *This is more of a presentation issue: the authors provide in luxury of detailed tables reporting the various comparisons, both in the main text at a supplementary material. However, is the number of comparisons is so great that for the reader it becomes very cumbersome and unwieldy to be able to discern what was actually found and what significant improvements are worthy of focus. A summary table might be a good way to make it easier for the reader to understand what was most relevant and most important.*

Following the reviewer's recommendations, we have created a supplementary table to summarize only the statistically significant changes found in the intermediate and final outcomes of the interventions in each country (Table S6).

5. *Lines 178-194 belong better in the introduction*

We have moved the description of the conceptual framework to the introduction section to make it more visible (line 138, page 5).

6. *Line 176: "This study is oriented by a comprehensive conceptual framework and care coordination of cross care levels..." To what framework are you referring? Please specify.*

Our study is based on a previously developed conceptual framework for the analysis of care coordination across care levels, which comprehensively addresses the different types and dimensions of clinical coordination, the care coordination mechanisms to improve them, and the factors that influence care coordination (best described in Vázquez et al. (2015)(10)). This theoretical framework was initially developed based on an extensive literature review (11–13) and has been improved over the years through its application in a number of quantitative and qualitative studies in Latin America and Catalonia (Spain)(4,14–17).

Some of the main elements of this framework are summarized in the introduction section of this article (definitions and types of care coordination, types of care coordination mechanisms and factors). In order to clarify this further, a subtitle has been added within this section, as well as a sentence explaining the conceptual framework and the corresponding references (line 138, page 5).

1.	Vargas I, Vázquez ML. Understanding the factors influencing the implementation of participatory interventions to improve care coordination. An analytical framework based on an evaluation in Latin America. Heal Policy Plan Approv. 2020;

2.	Vargas I, Eguiguren P, Mogollón-Pérez A-S, Samico I, Bertolotto F, López-Vázquez J, et al. Can care coordination across levels be improved through the implementation of participatory action research interventions? Outcomes and conditions for sustaining changes in five Latin American countries. BMC Health Serv Res. 2020;20(1):941.

3.	Vazquez ML, Vargas I, Unger JP, De Paepe P, Mogollon-Perez AS, Samico I, et al. Evaluating the effectiveness of care integration strategies in different healthcare systems in Latin America: the EQUITY-LA II quasi-experimental study protocol. BMJ Open [Internet]. 2015;5(7):e007037. Available from: http://www.ncbi.nlm.nih.gov/pubmed/26231753

4.	Vazquez ML, Vargas I, Garcia-Subirats I, Unger J-PP, de Paepe P, Mogollón-Pérez AS, et al. Doctors’ experience of coordination across care levels and associated factors. A cross-sectional study in public healthcare networks of six Latin American countries. Soc Sci Med. 2017;182:10–9.

5.	Vargas I, Vázquez M-LL, Eguiguren P, Mogollón-Pérez A-S, Bertolotto F, Samico I, et al. Understanding the factors influencing the implementation of participatory interventions to improve care coordination. An analytical framework based on an evaluation in Latin America. Health Policy Plan. 2020;35(8):962–72.

6.	Vargas I, Eguiguren P, Mogollón A, López J, Samico I, Bertolotto F, et al. [Participatory design of interventions to improve clinical coordination in Latin America]. Gac Sanit. 2019;33(SC).

7.	Vargas I, Garcia-Subirats I, Mogollón-Pérez A-S, Ferreira-de-Medeiros-Mendes M, Eguiguren P, Cisneros A-I, et al. Understanding communication breakdown in the outpatient referral process in Latin America: a cross-sectional study on the use of clinical correspondence in public healthcare networks of six countries. Health Policy Plan [Internet]. 2018;czy016–czy016. Available from: http://dx.doi.org/10.1093/heapol/czy016

8.	Curran GM, Bauer M, Mittman B, Pyne JM, Stetler C. Effectiveness-implementation hybrid designs: combining elements of clinical effectiveness and implementation research to enhance public health impact. Med Care [Internet]. 2012;50(3):217–26. Available from: http://www.ncbi.nlm.nih.gov/pubmed/22310560

9.	Damschroder LJ, Aron DC, Keith RE, Kirsh SR, Alexander JA, Lowery JC. Fostering implementation of health services research findings into practice: A consolidated framework for advancing implementation science. Implement Sci. 2009;4(1).

10.	Vázquez ML, Vargas I, Unger JP, De Paepe P, Mogollón-Pérez AS, Samico I, et al. Evaluating the effectiveness of care integration strategies in different healthcare systems in Latin America: The EQUITY-LA II quasi-experimental study protocol. BMJ Open. 2015;5(7):1–10.

11.	Vázquez ML, Vargas I, Unger JP, Mogollón AS, Ferreira da Silva M, De Paepe P. Integrated healthcare network in Latin America: towards a conceptual framework for analysis. Pan Am J Public Heal. 2009;26(4):360–7.

12.	Terraza-Núñez R, Vargas I, Vázquez ML. [Coordination among healthcare levels: systematization of tools and measures]. Gac Sanit [Internet]. 2006;20(6):485–95. Available from: http://www.ncbi.nlm.nih.gov/pubmed/17198628

13.	Vázquez Navarrete ML, Vargas Lorenzo I, Mogollón-Pérez AS, Ferreira da Silva MR, Unger J-P, de Paepe P. Redes integradas de servicios de salud en Colombia y Brasil: Estudio de casos. Editorial Universidad del Rosario; 2018.

14.	Vargas I, Mogollón-Pérez AS, De Paepe P, Ferreira Da Silva MR, Unger JP, Vázquez ML. Barriers to healthcare coordination in market-based and decentralized public health systems: A qualitative study in healthcare networks of Colombia and Brazil. Health Policy Plan. 2016;

15.	Esteve-Matalí L, Vargas I, Sánchez E, Ramon I, Plaja P, Vázquez M. Do primary and secondary care doctors have a different experience and perception of cross-level clinical coordination? Results of a cross-sectional study in the Catalan National Health System (Spain). BMC Fam Pract. 2020;21(1).

16.	Aller MB, Vargas I, Coderch J, Calero S, Cots F, Abizanda M, et al. Doctors’ opinions on clinical coordination between primary and secondary care in the Catalan healthcare system. Gac Sanit. 2019;33(1):66–73.

17.	Aller MB, Vargas I, Coderch J, Calero S, Cots F, Abizanda M, et al. Development and testing of indicators to measure coordination of clinical information and management across levels of care. BMC Health Serv Res. 2015;
