# Supplementary material for: Evaluating the effectiveness of care coordination interventions designed and implemented through a participatory action research process: Lessons learned from a quasi-experimental study in public healthcare networks in Latin America
Source: PLoS One. 2022 Jan 12;17(1):e0261604. doi: 10.1371/journal.pone.0261604 (PMC8754346; doi:10.1371/journal.pone.0261604)
Supplement: Attachment — Submitted filename: Reviewer Comments.docx [file pone.0261604.s011.docx]

Thank you for the opportunity to review this manuscript that describes one piece of a very ambitious project. The aim of this study was to evaluate the effectiveness of PAR interventions in improving clinical coordination between care levels.

This paper has already been through one round of reviews and I am joining as a reviewer in the second round. Unfortunately, I must highlight issues that really need to be addressed, that were not highlighted in the first round.

First, I want to congratulate the whole team for this large project that spanned five (actually, six) countries using a challenging, complex intervention (PAR) with complex methods. There is a LOT to cover in this paper and the authors have clearly improved the paper from the first round. However, key issues must be addressed. These suggestions will help make this paper much clearer and should help strengthen the framing of results by being more transparent about methods and rationale. One note: it was difficult for me to track responses to prior reviews because line and page numbers did not match the content described in each response.

MAJOR REVISIONS

1. This is an easy fix but a major problem: the authors use the term “intervention” throughout. At times “intervention” seems to refer to the PAR approach used and other times, it seems to refer to the “solution” chosen by each country. If my understanding is accurate, I suggest this terminology:
   a. Identified gap/problem: this is the “problematized” issue identified by baseline data(?) [line 165] within each IN/country
   b. Chosen solution: this is the “selected intervention(s)” [line 165] within the INs
   c. PAR Approach: three general approaches were used by the five countries [lines 166-170] – but each country followed very different PAR approaches that don’t seem to align with the 3 types (ref Table S1).
   d. *Note, throughout, five countries are referenced but in Line 185, six countries are referenced. At Line 170, it states that Argentina was dropped because the “selected intervention” was not implemented. The authors need to provide better justification of dropping this country. Using an “intent to treat” approach, Argentina should be included. However, the authors could frame this analysis as including only “completers” or “active participants.” Either way, the failure of the Argentinian IN to implement their chosen intervention (aka chosen solution) needs to be discussed in limitations. Previously published findings all seem to have included all six countries.*
2. In response to the first item from Reviewer #2, the authors present a nice summary of previously published findings that maps out the work already done and how it informs the current manuscript. I suggest reworking the Introduction to better walk readers through what already has been reported and what this manuscript adds. The basic story arc seems to be this: you’ve reported on the PAR approaches, the “problems/gaps” and “solutions” chosen by each IN within each country and now, your aim is “to evaluate the effectiveness of PAR interventions in improving clinical coordination between care levels.”
   a. It would be more accurate to state that your aim is “to evaluate the effectiveness of PAR approaches on *indicators (or perceptions)* of clinical coordination between care levels.” 🡪 you are reporting perceptions/self-reported measures via the COORDENA survey instrument, not actual measures of “coordinated care.”
   b. Consider moving Table S1 to the main manuscript. By restructuring this table, it can quickly “tell the story” of each country. Here’s a suggested layout:
      i. Title 🡪 Table 1: Description of Problems, Solutions, and PAR Approaches used by Country
      ii. The current Table S1 has this footnote: “More details on the characteristics of each intervention in: <http://www.equity-la.eu/en/publicaciones.php?t=PR>” but this link goes to presentations without a description of “interventions” (and consider noting that content is in Spanish)
      iii. Rows:
           1. Problem(s) (e.g., lack of coordination for diabetes patients)
           2. Solution(s) (e.g., create and implement guidelines shared by PC and SC providers that focus on essential practices and care pathways)
           3. PAR Approach – Joint Meetings:
              a. Description (e.g., Discuss mental health care cases face-to-face
              b. Who (e.g., PC teams and psychiatrists)
              c. Dose (e.g., 11 design(?) sessions with PC and SC providers)
              d. Duration (e.g., 7 months)
           4. PAR Approach – Virtual Consultations
              a. Description (e.g., Asynchronous virtual consultations in Mental Health via email)
              b. Who (e.g., PC doctors and psychiatrists)
              c. Dose (e.g., 11 consultations)
              d. Duration (e.g., 3 months)
           5. PAR approach – Other
              a. Description
              b. Who
              c. Dose
              d. Duration
      iv. Columns: list the five countries
   c. The text description of the 3 types of PAR used (in Lines 166-170) is confusing. Please clarify how these three general approaches align with the content of Table S1/new Table 1 outlined above.
3. The structure and content of all of the Tables needs to be reworked.
   a. Table 1 lists “demographic” descriptions of networks and providers within each of the five participating countries. This table is very similar to Table S2 and yet the numbers are different. This needs to be resolved.
   b. Table 1 is labeled as “Evaluation Survey (2017)” and yet the footnote in Table 1, for “***” and “**” says that these symbols indicate differences at baseline. These are for differences between IN and CN…at baseline? This Table should focus only on baseline differences, not a mix of both time points.
   c. Table 1 was suggested by a reviewer but there is no rationale for the choice of data to show in this table – e.g., sex and age of providers does not seem to have any relationship to the aim. I suggest moving this table to the Supplement except for a few of the rows:
      i. From Table 1, toward the bottom: I suggest listing “Enough time during consultation…” and “Knowledge of the Intervention” and “use of/participation in the intervention” be moved to a separate Table to present in Results. These lines are inferred in the Discussion when explaining possible reasons for differences and lack of differences in the care coordination indicators.
   d. Tables 2 and 3 are very confusing. Methods need to better describe what “PR” is and why that was chosen as your main outcome. AND provide rationale for showing both IN versus CN differences in Table 2 PLUS showing PRs for reversed order in time (2015 vs 2017). I’m seeing 100 paired comparisons across these two tables! With no adjustment for repeated comparisons, statistically. See below for more on Methods.
      i. This paper should be reviewed by a statistician and/or better framed to avoid reaching conclusions that are beyond what the data can support
4. The Methods Section needs to be significantly strengthened.
   a. Start with the description of your care coordination framework.
      i. Readers need more information on the components of care coordination as conceptualized within the COORDENA questionnaire. Readers are not familiar with COORDENA. It seems that CC (care coordination) comprises 1) interactional factors, 2) Organizational Factors; 3) Coordination of information; 4) Consistency of care ; and 5) patient follow-up
         1. You conceptualize 1-2 as “intermediate” and 3-5 as “distal.” More accurate labels might be *determinants* of CC versus *perceptions* of CC…or other similar labeling
      ii. There is an implied causal/influential pathway: “Enough time during consultation…,” “Knowledge of the PAR intervention,” and “Use of/participation in PAR” (listed in Table 1) may influence Interactional and Organizational factors (listed in Tables 2-3) which may lead to (influence) Coordination of Information, Consistency of care, and Patient follow-up (listed in Tables 4-5). Please describe your hypothesized pathway and intent to explore associations along this pathway.
   b. Study Design
      i. You characterize this study as “quasi-experimental” which not a helpful label. It would be more accurate to describe this as a prospective non-randomized trial. You pre-selected INs and CNs and then tested the effects of PAR on perceptions of CC (care coordination). Each network identified different problems/gaps and solution(s) and each used a different mix of PAR approaches
   c. Study Population and Sample
      i. Your unit of analysis seems to be “Country (or Network(?))” – an intervention network (IN) and control network (CN), which are each in separate countries. State this.
      ii. Within each country/network, you sampled doctors at two levels: 1) primary; and 2) secondary. Define what types of care are included in each of these levels. These providers are clustered by network – this forms the basis for your outcomes.
   d. Primary outcome
      i. Hundreds of paired comparisons are presented. Any statistician (and I am not a statistician) will warn against multiple comparisons – and any conclusions based on a seeming random assortment of “significance” from among the hundreds.
      ii. A single primary analysis is needed. The detailed paired comparisons can be presented (in a more limited and thoughtful way) to help *explain* (negative?) results of your primary analysis.
      iii. Consider a table that shows baseline results for IN and CNs overall, with ranges across the countries.
      iv. What is “PR” – how is it computed? This seems to be your primary outcome. However, it is not used appropriately. A single set of ratios – or difference in difference scores? – should be listed for each of the measures
          1. Regarding measurement: will the COORDENA measures support “scale-level” averages for each of the 5 dimensions (e.g., organizational factors as a single averaged score, instead of comparing every item (e.g., a single indicator for organizational factors instead of 2 separate items))?
5. When the measures are better defined as suggested above, along with an appropriate analytic plan, write up results that follow that flow: present primary outcomes followed by exploratory analyses to help explain findings.
   a. Table S6 is a nice summary of “significant” findings but combining “significance” for IN vs CN comparison with baseline versus follow-up within IN-only, into the same table, is not theoretically justified.
